# Analyzing Receptor Assemblies in the Cell Membrane Using Fluorescence Anisotropy Imaging with TIRF Microscopy

**DOI:** 10.1371/journal.pone.0100526

**Published:** 2014-06-19

**Authors:** Miklos Erdelyi, Joseph Simon, Eric A. Barnard, Clemens F. Kaminski

**Affiliations:** 1 Department of Chemical Engineering and Biotechnology, University of Cambridge, Cambridge, United Kingdom; 2 Analytical Science Division, National Physical Laboratory, Teddington, Middlesex, United Kingdom; 3 Department of Pharmacology, University of Cambridge, Cambridge, United Kingdom; 4 Department of Optics and Quantum Electronics, University of Szeged, Szeged, Hungary; Julius-Maximilians-University Würzburg, Germany

## Abstract

Signaling within and between animal cells is controlled by the many receptor proteins in their membrane. They variously operate as trans-membrane monomers and homo- or hetero-dimers, and may assemble with ion-channels: analyses thereof are needed in studies of receptor actions in tissue physiology and pathology. Interactions between membrane proteins are detectable when pre-labeled with fluorophores, but a much fuller analysis is achievable via advanced optical techniques on living cells. In this context, the measurement of polarization anisotropy in the emitted fluorescence has been the least exploited. Here we demonstrate its methodology and particular advantages in the study of receptor protein assembly. Through excitation in both TIRF and EPI fluorescence illumination modes we are able to quantify and suppress contributions to the signal from extraneous intra-cellular fluorescence, and we show that the loss of fluorescence-polarization measured in membrane proteins reports on receptor protein assembly in real time. Receptor monomers and homo-dimers in the cell membrane can be analyzed quantitatively and for homo-dimers only a single fluorescent marker is needed, thus suppressing ambiguities that arise in alternative assays, which require multiple label moieties and which are thus subject to stoichiometric uncertainty.

## Introduction

The identification and analysis of specific protein-protein interactions in vertebrate cell membranes is now a major need in cell physiology and pathology. Examples of its potential importance include the dimer formation deduced for many G-protein-coupled receptors (GPCRs) [1]–[3], or the functional complexes between some GPCRs and specific ion-channels [4]–[6]. Potentially a large number and variety of membrane protein types could be involved in such interactions, activated by locally-released specific agonist molecules. The analysis of these cases within living cells clearly requires refined optical methods and a range of optical tools is now available for this purpose. Most are based upon various exploitations of the Förster Resonance Energy Transfer (FRET) between donor and acceptor fluorescent labels on the protein targets. The labels currently in use thereon are usually a pair of complementary fluorescent proteins pre-attached to the target protein via DNA manipulation and co-expression. In such analyses on living cells the most commonly used method at present is ratiometric, measuring the sensitized emission [7], which is produced in FRET at the acceptor fluorophore (seFRET). This method is compatible with commonly available microscopy platforms and offers good temporal resolution for the investigation of highly dynamic phenomena in living cells. However, it requires extensive corrections and control measurements to account for the unequal intra-cellular distributions of donor and acceptor labels and for fluorescence cross-excitation [8]. Proper calibration of seFRET requires a model polypeptide construct linking in tandem the two fluorophores used, and even then the method is often only semi-quantitative in nature. A second method applicable in such doubly-labeled cells is to register the decrease of fluorescent lifetime of the donor emission, caused by FRET between donors and acceptors (fluorescence lifetime imaging microscopy, FLIM) [2], [9]. That method has a poor photon economy, and acquisition speeds are too slow to detect phenomena of a highly dynamic nature [10]. Strictly speaking the technique requires an overabundance of acceptor fluorophores to be quantitative, and there are restrictions on the number of suitable donor fluorophores with the required characteristics [9]. A different approach, which avoids this problem, is based on the so called number and brightness analysis (NBA), which requires only a single fluorophore moiety for labeling [11]–[12]. It is related to fluorescence correlation spectroscopy and monitors fluctuations in signal intensity to differentiate between mobile and immobile fluorophore fractions and cluster size. The technique is mathematically complex, and requires use of a diffusion model and good signal to noise ratio to quantify brightness fluctuations from the tracked particles. The technique has been successfully used to study receptor dimerization for example in [13] where fluorescence fluctuations in quantum dot labels were used to track the dimerization of single EGFR receptors. Single molecule photobleaching [14]–[16] and super-resolution [17] techniques have also been successfully applied to follow receptor protein dimerization and oligomerization in fixed and even live cells. However, the latter techniques require proteins to be present at low expression levels in order to keep the point spread functions generated by individual fluorophore labels spatially separated in the recorded images.

A different approach, which is simpler to implement and interpret, and which, similarly to NBA, only requires a single fluorophore moiety for labeling, is fluorescence anisotropy imaging microscopy (FAIM) [18]. FAIM measures energy transfer, homo-FRET, occurring between identical and proximate fluorophores. Steady-state FAIM (ssFAIM) quantifies the decrease in the polarization of the emitted fluorescence light (anisotropy) produced by homo-FRET [19], and has recently been adopted for the study of protein self-assembly reactions [20], [21]. Homo-FRET is possible between identical fluorophores if their excitation and emission spectra exhibit a degree of overlap, which is the case for the majority of biological fluorophores [22], [23]. This fundamental feature simplifies the labeling protocols and, as is the case for NBA, significantly eliminates artifacts which can result from differences in local expression levels of labeled donors and acceptors. Another unique feature of ssFAIM is that, over the range of signal intensities used in studies such as the present one, it is independent of the concentration and molecular brightness of the fluorescent protein in the field of view during measurement.

There is a general problem, however, in measuring FRET by any technique between membrane proteins of living cells, after their usual expression in a host cell by transfection in the pre-labeled form. This problem arises because much of the fluorescent protein generally remains in the cell interior and may also be present there from the trafficking of the labeled receptors or their additional internal compartmentalization. The diffuse optical overlap of intracellular proteins and membrane-bound fractions of labeled proteins cannot in practice be efficiently distinguished, and this reduces accuracy. Here we present a method based on FAIM, which overcomes this problem by using wide-field fluorescence optics combined with illumination modes alternating between EPI fluorescence and total internal reflection fluorescence microscopy (TIRFM) [24]. The method distinguishes sufficiently the membrane-bound from the intra-cellular fractions of a given labeled protein. Here we rigorously validate and describe this FAIM/TIRF method and illustrate how it can be applied to a biologically important topic, the dimerization of GPCRs in the plasma membranes of living cells.

The GPCRs constitute by far the largest group of trans-membrane receptor proteins in vertebrates [25]. Many of them control ion channels and other effectors of signaling in cell membranes generally [3]–[6]. We analyze here typical membrane protein associations of the P2YR type, a member of the majority Class A of GPCRs. P2YRs are a family of 8 receptors for signaling nucleotides, known since 1993, which is exceptional in having members occurring in the cell membrane of every type of vertebrate cell so far investigated [26]. Also, its members are activated by ATP or related small nucleotides, which are ubiquitous native agonists for them. For these reasons its study in cell membranes is convenient for biophysical studies and relevant to a variety of biological functions and disorders. We obtained evidence by earlier FRET-based methods to suggest that P2Y1R and P2Y2R can each readily form non-covalently-linked stable dimers [3]. Importantly, some GPCRs can vary their signaling functions by such dimerization [1], [2]. The quantitative determination of the dimer and monomer fractions of such a receptor within the cell membrane is therefore needed. We use this example to demonstrate improved methods for this. We address the influence of microscope optics on the quantitation of measured anisotropies and give practical guidelines to achieve optimal measurement performance, with relevance not only to the study of receptor proteins such as the P2YR family but to membrane protein biophysics in general.

## Materials and Methods

### Total Internal Reflection Microscopy

In total internal reflection (TIRF) microscopy a thin layer close to the coverslip/sample interface is illuminated via a collimated beam impinging on the coverslip at an angle of incidence larger than the critical angle [24]. The beam is totally reflected and only the evanescent field with a penetration depth of approximately 100 nm excites the sample in the case of a glass-water interface and an excitation wavelength of 450 nm [24]. This illumination method is ideal for imaging thin layers, such as the plasma membrane of a cell, with minimum background fluorescence, and hence for spatially distinguishing intracellular processes from those occurring in the plasma membrane. There are two practical modes of TIRF illumination, which are referred to as prism and objective based TIRF geometries. In the following we focus on objective based TIRF illumination, which is a more practical configuration for many types of biological experiment.

**Figure 1:**
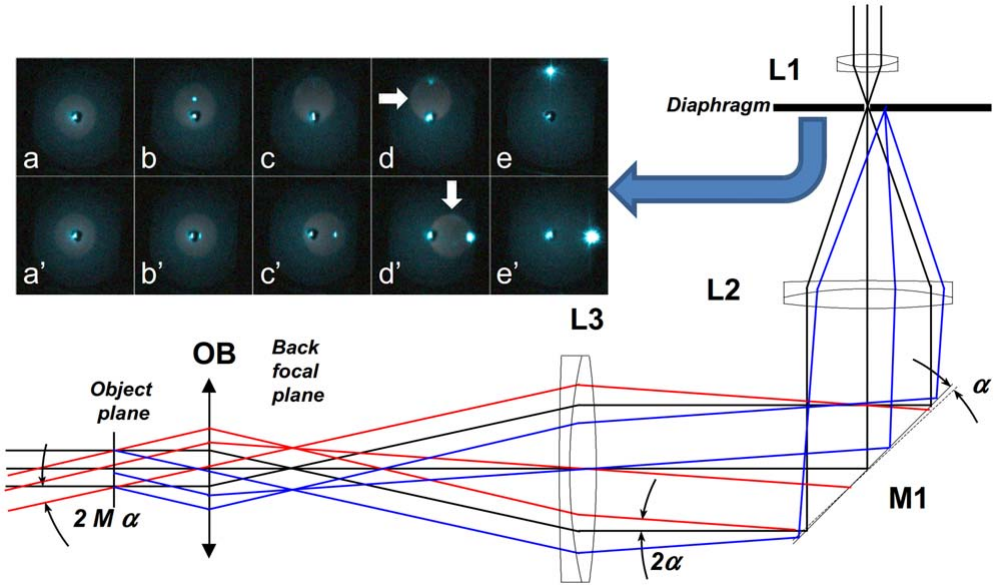
Excitation beam path for anisotropy measurement of receptor dimerization. In EPI illumination (black rays) the excitation beam is perpendicular to the sample. The angle of inclination on the sample can be set by mirror M1. Under a highly inclined illumination mode, the excitation beam (red) is partially back reflected (blue) and can be captured on the diaphragm at different (M1) mirror positions and under *p-* (a–e) and *s*-polarizations (a’–e’).

A schematic view of the excitation beam path is depicted in Figure 1. The beam is expanded (via lenses L1 and L2) and focused (via lens L3) into the back focal-plane of the microscope objective (OB). The optical elements form a relay system, so that the angle of illumination of the sample can be controlled via tilting mirror M1, inserted into the common focal plane of lenses L2 and L3. In the EPI illumination mode (black rays) the sample is illuminated perpendicularly to the plane defined by the cover slip. In the applied configuration off-axis illumination (red rays) of the sample was obtained by tilting mirror M1. When a high-NA TIRF objective is used, the off-axis angle (2Mα, where M is the overall magnification of the objective and L3 focusing lens, and α is the tilt angle of the mirror) of the output beam should be higher than the critical angle for the glass-water interface (62°). The beam reflects back from the interface, and only the evanescent field penetrates into the sample and excites it. The beam reflects back partially (in the highly-inclined mode), or totally (TIRF mode) on the coverslip/sample interface. Proper alignment is easily checked *via* the back-reflected beam (blue rays), which can be captured and monitored on a diaphragm placed into the common focal plane of the telescope lenses (see inset images in Figure 1). The larger the tilt angle (α) of mirror M1, the larger the angle of inclination on the sample and also the off-axis position of the back-reflected beam on the diaphragm. The inset images show the back-reflected beam, at different mirror positions and for different polarization conditions. The top row depicts the case when the mirror was tilted so that the sample would be illuminated by a *p*-polarized beam. In the EPI mode (Figure 1 a) the beam reflects back directly. If the angle of incidence is increased, the intensity of the back-reflected beam slightly decreases, and then disappears when the angle of incidence is equal to the Brewster angle (Figure 1 c). When the angle of incidence is further increased (Figure 1, d–e), the intensity increases and reaches its maximum in the TIRF mode. When the mirror is tilted around the other axis, the sample is illuminated via an *s-*polarized beam. In this case the intensity of the back-reflected beam is increasing continuously with the angle of incidence. When the excitation filter is removed and a bright fluorescent sample (e.g. dye solution) is used, the fluorescence light can also be visualized in this plane as a weak halo (marked with white arrows). In the TIRF mode the intensity of the back-reflected beam increases (Figure 1, e and e’) and at the same time the intensity of the collected fluorescence light drops, since the excited volume decreases significantly. By monitoring the back-reflected light, it is possible to adjust precisely the polarization conditions and the angle of illumination. The optical arrangement shown in Figure 1 provides for facile switching between the TIRF and EPI modes, which is essential for the technique described here.

### Fluorescence Anisotropy

The fluorescence anisotropy, r is defined as
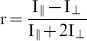
(1)where I_∥_ and I_⊥_ are the parallel and perpendicular components of the emitted light relative to the polarization of the excitation light. The value of anisotropy expresses the depolarization of light emitted from a molecule relative to the incident linearly-polarized excitation light. For static and non-interacting monomeric fluorophores the theoretically attainable maximum anisotropy value is *r = *0.4, which corresponds to I_∥_ *= *3I_⊥_. However, in biological analyses where excitation is made of a GFP-family fluorescent protein attached to a target receptor protein in an inhomogeneous cellular environment, significant rotational diffusion of the emitter occurs and in consequence the value of *r* is decreased [22]. There are additional effects leading to anisotropy loss, such as depolarization introduced by the complex optical pathway, an angular deviation of absorption and emission dipole axes in GFP. As a result the maximum *r* value seen in practice is around ∼0.30 or less in cell; here we measured it to be 0.24.

**Figure 2:**
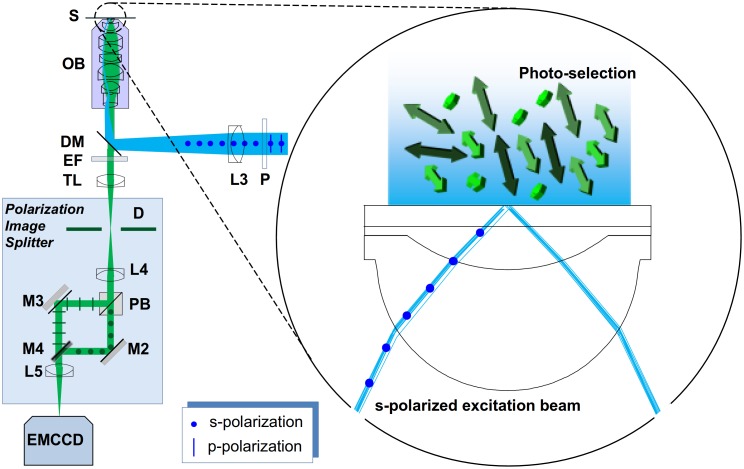
Steady-state fluorescent anisotropy measurement system. (P: polarizer, L3: focusing lens, DM: dichroic mirror, OB: microscope objective, S: sample, EF: emission filter, TL: tube lens, D: field stop, L4 and L5: imaging lenses, PB: polarization beam splitter, M2 and M3: mirrors, M4: D-shaped mirror).

The schematic view of the applied polarization sensitive detection can be seen in Figure 2. The emitted fluorescence light is collected and passed through the emission filter (EF). The polarization image splitter separates the parallel (||) and perpendicular (⊥) polarization components, and images them onto spatially-distinct zones of the CCD sensor. The linearly polarized excitation beam (blue) is focused onto the back focal plane of the microscope objective and illuminates the sample either in EPI or TIRF modes, as described in Figure 1. Here, the arrows represent the randomly oriented dipole moments for the individual fluorophores, which are excited via an s-polarized beam (polarization perpendicular to the figure plane). With respect to the figure plane these will preferentially excite molecules with dipoles oriented in a perpendicular direction as indicated by the lighter green colour in Figure 2.

### G-factor

The two polarization states of the emitted light are typically measured on two similar, but separate, detectors. To account for sensitivity differences Eq. 1 must be modified to:
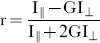
(2)where G-factor, G, is the ratio of the detection sensitivities of the detectors. In wide-field anisotropy measurements the G-factor is a function of pixel coordinates. Also, small optical path differences and aberrations introduced by the image splitter cause the images generated by the parallel (||) and perpendicular (⊥) polarization components to be different, and the co-ordinates in the two image channels must be mapped onto one another (image registration). For registration, either a structured sample or the aperture (field stop) used for cropping the field of view (D in Figure 2) can be used to establish reference and target co-ordinates for the mapping algorithm. The spatial dependence of the G factor can be measured using a highly diluted dye solution, with absorption/emission spectra similar to the sample to be measured. Assuming fast molecular rotation, and hence zero anisotropy, the intensity response of the two channels can thus be determined.

### Optical Depolarization

Anisotropy informs on the depolarization of the excited state dipoles. However, depolarization may arise from various causes, such as the rotation of the excited molecules between excitation and emission, homo and hetero-FRET, and optical depolarization effects. Separation of these effects requires additional calibration by independent methods. In this section we investigate the effect of the three most critical optical depolarization sources on the precision of the measured anisotropy values:

- Depolarization of the excitation beam caused by the high- numerical aperture (NA) objective.
- Depolarization of the emitted light caused by the high-NA objective.
- Polarization mixing caused by the finite extinction ratio of the analyzer.

**Figure 3:**
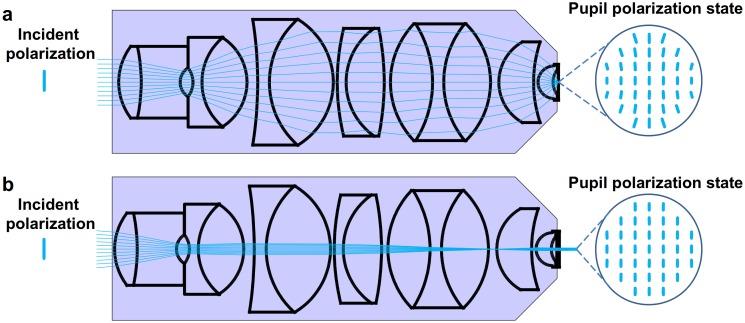
Depolarization introduced by a high NA microscope objective. 3(a) The microscope objective is illuminated by a linearly polarized plane wave and focuses the beam onto the sample. This mode of illumination is used in confocal microscopy. (b) The linearly polarized beam is focused into the back-focal plane of the objective (widefield illumination). The sample is illuminated by a collimated plane wave. The “pupil” polarization states in the image planes are depicted using vertically polarized incident beams. Widefield illumination (b) leads to lower loss of polarization in the illumination field than focused beam illumination (a) as confirmed by optical ray tracing simulations.

The spatial resolution of microscope objectives scales with their numerical apertures (NA). However, objectives with NA>0.5 affect the polarization state of the incoming beam due to the multiple and relatively large angle refractions [27]. Figure 3 shows results from ray tracing simulations (OSLO, Optics Software for Layout and Optimization) [28] using a commercial TIRF lens [29] in focusing (i.e. confocal) and wide field (EPI) illumination modes. The insets depict the polarization states in the sample plane when the objective is illuminated by a vertically polarized beam. In the focusing mode the beam is visibly depolarized in the four corners of the field of view, whilst the polarization state remains almost unaffected in EPI-illumination. The quantitative evaluation of OSLO simulations predicts 10^−6^ and 10^−5^ polarization cross-talks for the EPI and TIRF modes, respectively. In the focusing mode, relevant for confocal microscopy, the cross-talk can exceed 15%. The almost complete disappearance of the back-reflected beam when the Brewster angle is approached is evident in Figure 1 and is experimental proof of the low polarization cross-talk predicted by the OSLO simulation [28] in the excitation path, a distinct advantage for the method presented here over confocal anisotropy measurements.

The depolarization of the emitted fluorescence light passing through the objective was also investigated using high-NA correction theory [30] and OSLO simulations [28]. We found a difference between the results: the high-NA correction method predicted 4.4% cross-talk, while the OSLO simulation predicted only 1.16%. We attribute the difference to multiple refracting components in the lens system: they are not taken into account by the high-NA correction theory, but play an important role in the minimization of polarization artifacts. This appears to be in agreement also with experimental results presented by Dix and Verkman [27]. Taking cross-talk into account the measured parallel and perpendicular intensity components I_||_ and I_⊥_ are given by
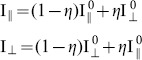
(3a–b)where *η* is the depolarization introduced by the objective, and 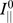, 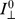 are the ‘true’ intensity components, assuming symmetrical cross-talk between the two channels and no loss of light. The corrected and measured anisotropy values r_0­_ and r, respectively, depend on the ratio of the parallel and perpendicular intensity components, respectively:
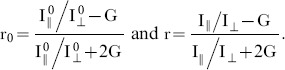
(4a–b)


Using these equations one can calculate the correction factor *f (r,η,G):*

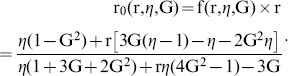
(5)The corrected anisotropy values were found to be 2.4–2.8% higher than the measured anisotropy values, for *G* = 1 and a depolarization value measured here to be *η* = 1.16%.

The extinction ratio (*ε*) of the analyzer (PB in Figure 2), affects the separation of parallel and perpendicular intensity components of the emitted light affects the measured anisotropy value. The measured parallel and perpendicular intensity components I_∥_ and I_⊥_ are
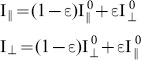
(6a–b)According to a similar derivation for anisotropy cross-talk the calculated relative error was found to be independent of the anisotropy, and to depend only on the extinction ratio. Therefore, an analyzer with an extinction ratio of 10^−3^ is sufficient to measure anisotropies with a precision of 0.3%.

As a conclusion we can summarize that the high NA microscope objective in the emission path is the major source of optical depolarization. However, the measured anisotropy values can be corrected based on the simulation results discussed above.

### Plasmid Constructs

Plasmids encoding the human P2Y1 receptor (hP2Y1R) protein, N-terminally tagged with the mTFP1 or the mTFP1-mTFP1 control dimer were constructed using standard molecular biological techniques. Briefly, the XhoI and BamHI fragment of the hP2Y1R coding sequence was inserted in-frame with the mTFP1 sequence into the pmTPF1-C plasmid (Allele Biotech, San Diego, CA, USA). The mTFP1 fluorescent protein coding sequence was also subcloned into pcDNA 4HisMax C vector (Life Technologies, Paisley, UK) and then linked in-frame through its 5′ end to the 3′ end of a second, identical mTFP1 sequence via a linker (60 bp) encoding the peptide: LEGQQMGRDLYDDDDKVPGS. The pECFP-18aa-EYFP tandem construct also used as a control has been described elsewhere [7].

### Choice of Fluorescent Proteins for Anisotropy Measurements

The enhanced green and yellow fluorescent proteins, mTFP1 [30] and EYFP [19] were used. This choice was made because those two proteins were recently found to make by far the best pair for FRET and FLIM experiments, out of 8 currently used monomeric fluorescent proteins tested in tandem polypeptide chain linkages [26].

### Tissue Culture and Transfection

Human embryonic kidney cells (HEK293T) were cultured in Dulbecco’s modified Eagle’s medium (DMEM) supplemented with 10% foetal calf serum (FBS) and antibiotics, penicillin and streptomycin (100 U/ml each, Life Technologies, Paisley, UK). The cultures were incubated in a humidified atmosphere with 5% CO_2_. The cells were initially plated on 22 mm coverslips coated with poly-L-lysine in 6-well plates. When the cells reached about 30% confluency they were transfected with the various plasmid constructs using lipofectamine 2000 (Life Technologies, Paisley, UK) [3]. 48 h after transfection the cells now transiently expressing the fluorescently labeled hP2Y1R or the control dimer constructs were used for live recording. Prior to the measurements, the cells on the coverslips were treated with 2U/ml apyrase (Sigma-Adrich, Gillingham, UK) for 4 h at 37°C to remove the nucleotides which are constantly endogeneously released in all such cells [3]. These coverslips were then lifted from the medium, briefly rinsed with Hank’s balanced salt solution (HBSS) containing 1 mM MgCl_2_ and 1 mM CaCl_2_, mounted in the recording chamber and overlaid either with HBSS containing 1 mM MgCl_2_ and 1 mM CaCl_2_, or with solutions containing P2Y1R-selective agonist or antagonists. The hP2Y1 receptors, N-terminally tagged with the mTFP1 fluorescent protein and heterologously expressed in HEK 293 cells on the mounted coverslips, were activated by incubation with 2-methylthio-ATP (2-MeS-ATP (10 µM)), a selective agonist [32], for the P2Y1 receptor, for 30 min at 37°C. The anisotropy measurements, both in epifluorescence (EPI) and in TIRF mode, were then made on live cells. The P2Y1 receptor activation by 2-MeS-ATP (Tocris, Bristol, UK) was completely blocked by the P2Y1R selective antagonist [33] 2-iodo-N^6^-methyl-(*N*)-methanocarba-2′-deoxyadenosine-3′, 5′-bisphosphate, MRS 2500 (Tocris, Bristol, UK; 1 µM, 30 min, 37°C).

## Results and Discussion

**Figure 4:**
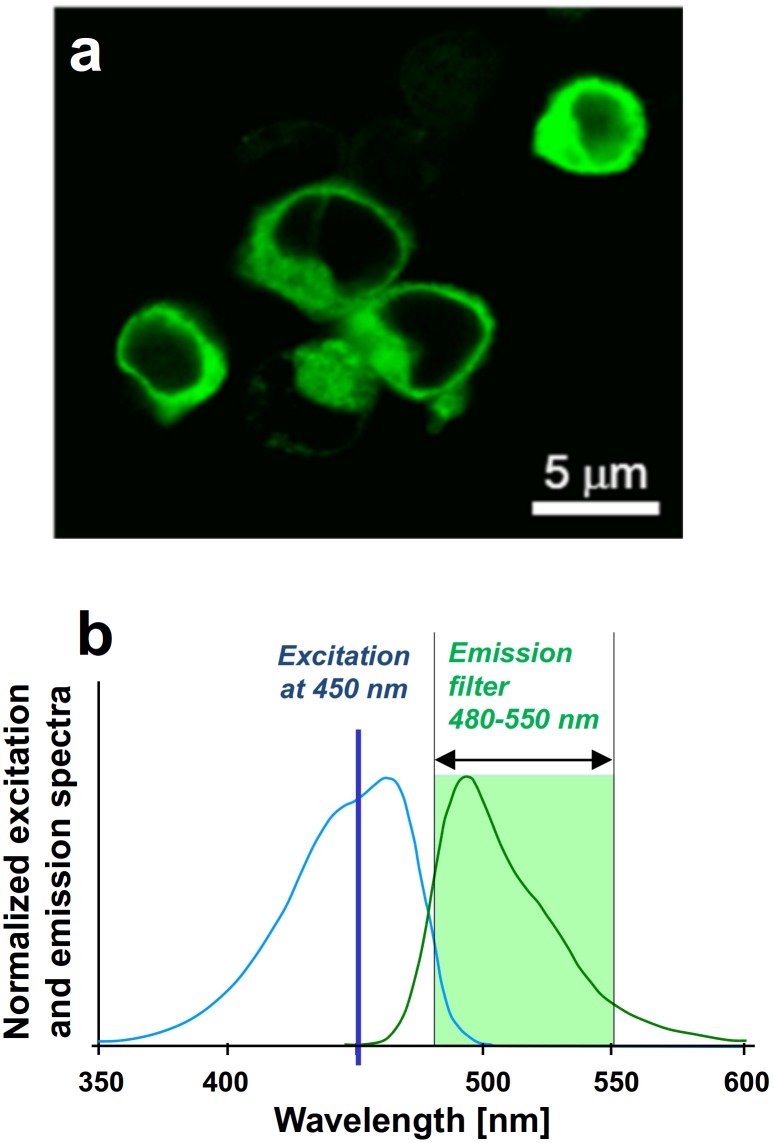
HEK 293T cells labeled by mTFP. (a) Confocal image of HEK 293T cells heterologously expressing the P2Y1 receptor protein, N-terminally tagged with mTFP1. (b) Normalized excitation and emission conditions used for mTFP1.

Living HEK293T cells on a coverslip were labeled by DNA transfection to express the P2Y1 receptor (P2Y1R) having the green-fluorescent protein mTFP1 attached. Figure 4, a–b show a confocal image of the labeled cells, and the normalized excitation and emission spectra of mTFP1 [31], [34], which was selected for its favorable properties for FRET-based analyses [9]. The DNA constructs were cloned to effect attachment of the label to the receptor N-terminus since that site is extracellular in GPCRs, where most GPCRs (including P2YRs) [3] have sensitive C-terminal tails carrying binding domains essential for interactions with other membrane proteins [35]. We have previously shown [3] that such protein attachment at the N-terminus of P2Y1R is favorable for imaging, and that this does not change the receptor protein’s functional properties. For calibration we performed anisotropy measurements in solutions containing either monomeric mTFP1 or mTFP1-tandem constructs, which mimic the response of monomeric and dimeric forms of mTFP1-tagged P2Y1R receptors, respectively.

The labeled cells were analyzed in the fluorescent anisotropy measurement system shown schematically in Figure 2. Laser light was used to excite the labeled cells but (as noted above) in such transfections there is always additional significant fluorescence in the cell interior from the excess of labeled receptors in the trafficking or in the compartmentalization routes into lysosomes, endosomes, etc., each of which can interfere considerably with optical measurements on the cell-membrane-bound receptors. We therefore measured ssFAIM in wide-field fluorescence optics in two alternative modes, one in total internal reflection fluorescence microscopy (TIRF) and another in EPI-fluorescence microscopy (EPI) across the same field of view.

In what follows we will show (i) how homo-FRET was verified to be the main contributor to observed anisotropy changes and, (ii), how the sequential recording in EPI and TIRF illumination modes permits the quantitative resolution of complex formation of membrane-bound proteins.

### Red-shifted Excitation

**Figure 5:**
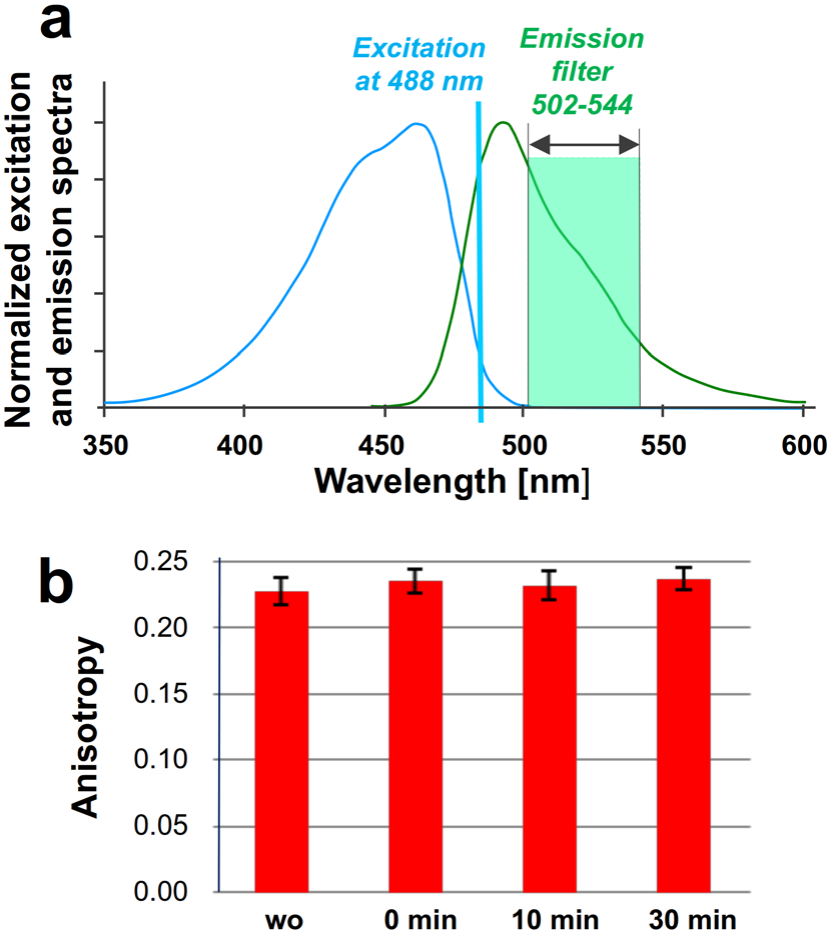
Red-edge excitation suppresses homo-FRET. (a) Excitation and emission spectra of mTFP1 with the applied excitation changed to the 488 nm laser line and with a narrow-range emission filter. In this situation, the red-edge detection suppresses homo-FRET when the spectral overlap between the excitation and emission spectra of mTFP1 is diminished. (b) Red-edge anisotropy measurement in the TIRF mode shows no loss of anisotropy upon agonist addition, demonstrating that the change of rotational diffusion is negligible, and the anisotropy change is the result of increased homo-FRET upon dimerization.

The extent of anisotropy measured for an excited fluorescent protein can be affected by its rotational diffusion (which would vary with the hydrodynamic radius of the molecule and the local viscosity), by energy transfer between the protein molecules and their environment (other molecules or surfaces), or by optical depolarization. To distinguish between rotational diffusion and homo-FRET upon dimer formation we excited the donor, mTFP1, at the extreme red edge of its absorption spectrum [26]. Red-edge excitation suppresses homo-FRET because the overlap between excitation and emission spectra of mTFP1 is greatly diminished (Figure 5a). Any residual anisotropy change observed can thus be attributed to rotational diffusion. Upon red-edge excitation at 488 nm of P2Y1 receptors N-terminally tagged with mTFP1, heterologously expressed in the membranes of HEK 293T cells, no anisotropy changes were observed (Figure 5b) both in the presence, and absence, of a 2Y1R -selective agonist, 2-MeSATP (10 µM). The measured anisotropy value did not change, proving that the main contributor to the anisotropy change is homo-FRET. The negligible rotational diffusion term can be explained by the relatively long rotational correlation time (23 ns for GFP) compared to the fluorescence lifetime (2.69 ns) of mTFP1 [34].

### Dimer Formation of P2Y1R Receptor Protein

**Figure 6:**
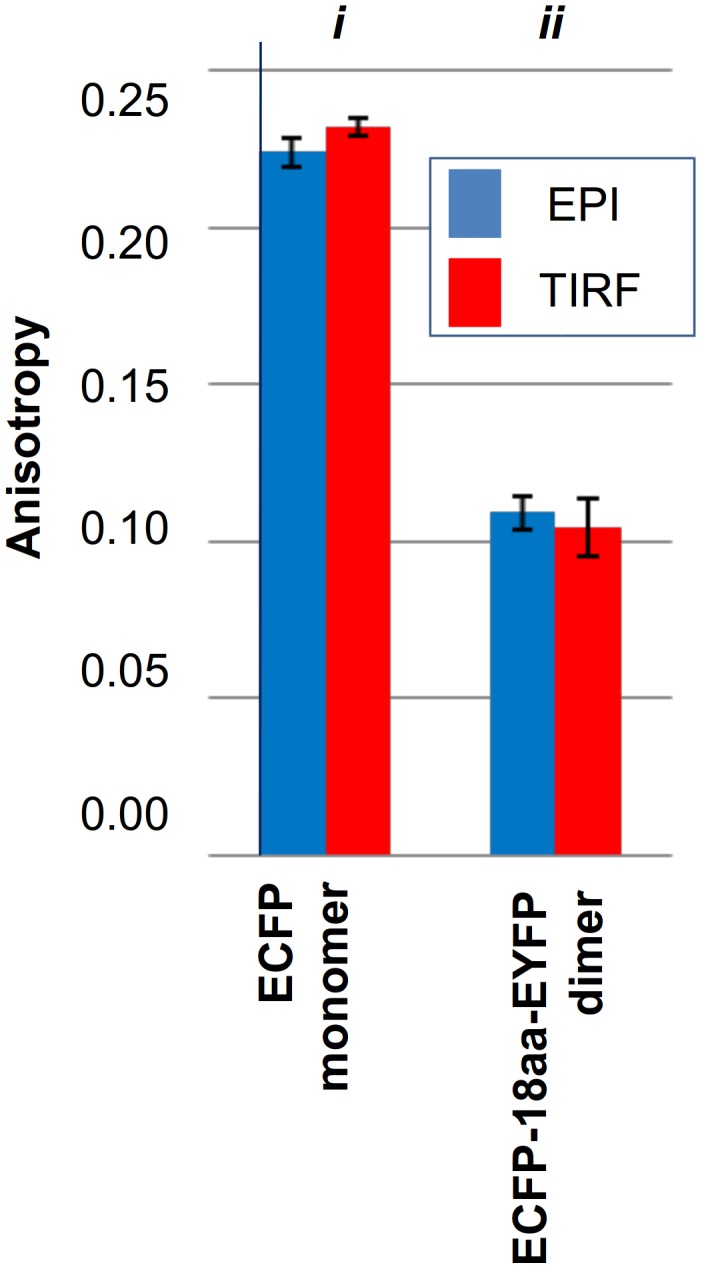
Measured anisotropy of ECFP monomers and ECFP-EYFP dimers. Measurements of anisotropy were made in the EPI or in TIRF mode, in cells expressing ECFP alone (i) or ECFP and EYFP sequences linked by an 18-aminoacid spacer (ii).

**Figure 7:**
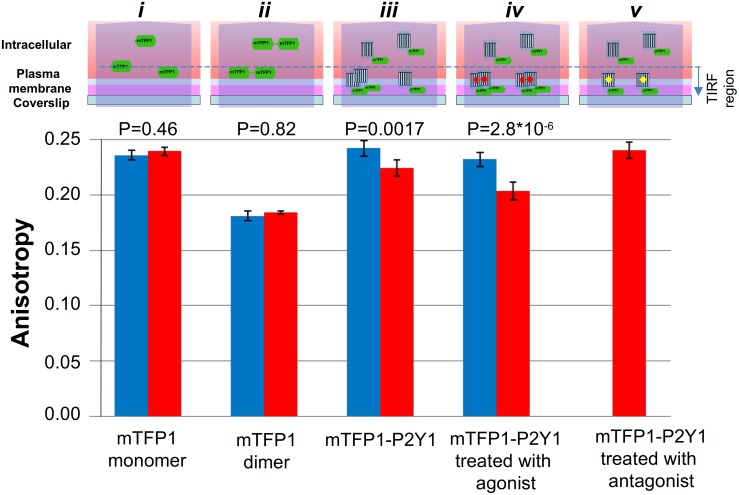
Measured anisotropy in different illumination modes, for analysis receptor assemblies in the cell membrane. Anisotropy, measured in the TIRF and EPI modes, of mTFP1 alone (i), or in a pair joined by a 20-aminoacid linker (ii), or attached to P2Y1R without (iii), or with (iv) the 10 µM2-MeSATP agonist or the 1 µM P2Y1R-selective antagonist MRS-2500 present (v). The diagrams above the bar plots represent the schematic interpretation of the anisotropy results in the TIRF and EPI illumination modes. (i) The monomeric mTFP1 fluorescent protein, expressed alone, localizes only in the interior of the cell, since it does not translocate into the plasma membrane or form dimers. The measured anisotropy value was found to be independent of the illumination modes. (ii) In control measurements with the mTFP1-20aa-mTFP1 construct, the mTFP1 homo-dimer is intracellularly located and showed much-reduced anisotropy due to homo-FRET between the two linked chromophores, under both illuminations. (iii) The P2Y1R was N-terminally tagged with mTFP1 and expressed. In the EPI mode the intracellular, monomeric mTFP-P2Y1R fusion protein produces the main component of the anisotropy, but in the TIRF mode its observed location is strongly restricted to sites within and near the membrane. (iv) Upon agonist treatment, the labeled P2Y1 receptors readily form dimers in the plasma membrane with almost 100% efficiency. (v) Application of a highly potent P2Y1R selective antagonist, completely reverses the agonist-induced decrease in anisotropy in the TIRF mode, confirming the P2Y1R involvement.

ssFAIM is capable to detect both homo- as well as hetero-FRET in contrast to the seFRET and FLIM methods, and we validated the method using monomeric and dimeric chimeras containing ECFP, EYFP and mTFP1 labels. Figure 6i and Figure 7i depict the measured anisotropy values for ECFP (*r* = 0.224±0.005) and mTFP1 (*r* = 0.236±0.004) monomers respectively, when heterologously expressed in HEK 293T cells. The monomeric mTFP1 fluorescent protein was expressed only inside the cell (Figure 6a) and did not form dimers. A reduced anisotropy was measured when the ECFP or the mTFP1 molecules were linked to EYFP and mTFP1 molecules, respectively, by polypeptide linkers of either 18- or 20-aminoacids in length (*r* = 0.109±0.005 and *r* = 0.181±0.004 in Figure 6ii and Figure 7ii). The measured anisotropy values were found to be independent of the illumination modes (blue and red correspond to EPI and TIRF illuminations in Figure 6 and 7), since all the constructs without the receptor proteins were expressed and localized only inside the cell and did not translocate to the plasma membrane.

This highlights the contribution of intracellular signals even under TIRF illumination since the depth of the evanescent field far exceeds the thickness of the membrane (∼100 nm penetration depth versus ∼10 nm thin membrane, respectively). Because receptor proteins are in their monomeric states in the cytoplasm but may be present in both monomeric and dimeric form in the plasma membrane the ssFAIM signals are complex mixtures from all three species, whose relative contribution to the observed signals depends on illumination mode (EPI vs TIRF). These effects are shown schematically above the bar plots in Figure 7, which shows data for mTFP1-P2Y1 fusions measured both in EPI and in TIRF illumination modes. It is seen that the measured anisotropy value for mTFP1-P2Y1 in EPI mode is essentially equal to the monomeric value measured for mTFP1 expressed alone (*r* = 0.242±0.007 in Figure 7iii). The contribution of constitutive dimers to the anisotropy is negligible in this state. This result is consistent with the fact that the sampling volume in EPI mode exceeds that of the TIRF measurements by a factor of four (600 nm depth of field in EPI illumination versus the 100 nm penetration depth in TIRF mode), so that the intracellular, and monomeric fraction of P2Y1R completely dominates the ssFAIM signal over the membrane contribution and demonstrates that receptor fusion does not significantly alter the fluorescent properties of mTFP1. A small, but significant reduction in *r* is seen in going from EPI to TIRF illumination (*r* = 0.224±0.007, Figure 7iii) demonstrating that in the membrane a non-negligible fraction of mTFP1-P2Y1 exists in dimeric form. Next we measured the anisotropy values of mTFP1-P2Y1 before and after the removal of the endogeneous nucleotides by apyrase treatment, or after activation of the P2Y1 receptors by their agonist (Figure 7iv). The treatment resulted in significant and reproducible loss of anisotropy measured with TIRF illumination modes indicative of increasing levels of dimer formation. A p-value analysis (see Figure 7) confirmed the capability of the technique to distinguish monomeric and dimeric fractions of the receptor on the membrane, and the distinction of membrane bound and internal protein. For mTFP1 and the mTFP1 tandem control constructs both TIRF and EPI data are indistinguishable (p>0.4, using EPI data as the null hypothesis). However, for mTFP2-P2Y1 treated without, and with, agonist the differences between TIRF and EPI data are statistically highly significant (p<<0.002). This confirms not only that membrane and non-membrane bound protein can be distinguished, but also the native and agonist induced dimer fraction.

Based on our previous study by other methods [3] we can assume that upon agonist treatment P2Y1 receptors form dimers in the plasma membrane with almost 100% efficiency. The difference between the measured anisotropy of the mTFP-20aa-mTFP control (Figure 7ii) and agonist treated sample can be explained by the stray excitation of intracellular monomeric P2Y1-mTFP constructs. Assuming a linear dependence of anisotropy on the monomer/dimer ratio:
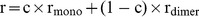
(7)Here c is the fraction of monomers contributing to the signal (both intracellular and membrane bound) and 1-c is the fraction of dimers, which according to previous discussion, is only present on the membrane. Using this analysis, and the fact that agonist treatment leads to 100% dimers on the membrane, we can estimate the fraction of constitutive dimers in the cell membrane, i.e. before agonist treatment, to be 45±20%, where the error denotes variations from cell to cell. We note that the apyrase treatment did not affect the dimer/monomer ratio significantly suggesting that only a small number of receptor dimers had been formed via activation by endogenously released ATP. Finally, application of a P2Y1 receptor selective antagonist, MRS 2500, completely reversed the 2MeSATP-induced decrease in anisotropy in TIRF mode (Figure 7v), confirming the P2Y1 involvement. The results confirmed that mTFP1 is an excellent reporter for receptor dimerization and that the developed technique, FAIM combined with TIRF microscopy, is of general applicability in the study of membrane protein associations.
